# Bacteriostatic Potency of Fe_2_O_3_ Against *Enterococcus faecalis* in Synergy with Antibiotics by DDST Method

**Published:** 2019

**Authors:** Erfan Shahbazi, Firouzeh Morshedzadeh, Davood Zaeifi

**Affiliations:** 1. Department of Microbiology, Shahid Beheshti University, Tehran, Iran; 2. Department of Cell and Molecular Biology, University of Tehran, Tehran, Iran; 3. Department of Biology, Tehran North Branch, Islamic Azad University, Tehran, Iran

**Keywords:** *Enterococcus faecalis*, Ferric oxide, Nanopaticles

## Abstract

**Background:**

In this study, bacteriostatic potency of the Iron oxide nanoparticles against *Enterococcus faecalis* (*E. faecalis)* (a clinical sample and the ATCC11700 strain) was investigated.

**Methods:**

Nanoparticles’ bacteriostatic concentration was determined and used to appraise the characteristics of the Iron Oxide (Fe_2_O_3_) against the isolates. Antimicrobial examinations with 10^8 ^*cfu.ml*^−1^ were performed at the baseline. Due to evaluation level of potency, after performing Minimum Inhibitory Concentration (MIC), the assessment of death kinetic and susceptibility constant of nanoparticles was done by suspension at two MICs in 0 to 360 *min* treatment time.

**Results:**

Fe_2_O_3_ nanoparticles in size range of 10–50 *nm* demonstrated the most effective susceptibility reaction against *E. faecalis* and ATCC11700 strain in Z=78.125 *ml/μg*^ −1^ and 39.0625 *ml/μg*^ −1^, respectively. The kinetic reaction of *E. faecalis* against Fe_2_O_3_ suspension was supposed to be decreased through the elapse of treatment time, whereas increased concentration was along with bacteria growth after a certain time. So, the efficient concentration of nanoparticles was applied with semi-sensitive and antibiotic resistant for both strains. However, synergism of Fe_2_O_3_ nanoparticles with Ceftazidime and Clindamycin revealed a higher susceptibility compared with Fe_2_O_3_nanoparticles alone against *E. faecalis*.

**Conclusion:**

The experimental results reveal that Fe_2_O_3_ has a strong antimicrobial effect at a certain concentration over the time so could potentially be used for bacterial inhibition and this feature will be strengthened in combination with antibiotics.

## Introduction

Enterococci are one of the most frequent causes of nosocomial infections in the intensive care unit; they appear in this sector along with clumsily use of cephalosporins and other antibiotics to which enterococci are resistant. These bacteria are contagious and usually cause infection in the urinary tract, wound, bile duct, and blood at the hospital; also, they can cause meningitis in children and endocarditis in adults 1,2.

*Enterococcus faecalis* (*E. faecalis*) is one of the most common species of *enterococcus* which causes 85 to 90% of enterococcal infections. Gram-positive bacteria were previously classified as Group D *Streptococcus*, due to specific antigen which is teichoic acid. Most of these bacteria are non-hemolytic and sometimes are alpha-hemolytic which can be found in natural intestinal flora 1–4.

These bacteria are resistant to many antibiotics like Meropenem, Gentamicin, Ceftriaxone, Ceftazidime, Cefixime, Trimethoprim/Sulfamethoxazole, Erythromycin, and Clindamycin with above 60% resistance frequency rate and sensitivity to Vancomycin, Teico-planin, and Nitrofurantion based on report 3–9.

Iron oxide due to its biocompatibility and magnetic feature has been widely used in biomedical research 10,11. Nanoparticles (NP) of Iron oxide with certain sizes (almost less than 100 *nm*), are applied for targeted drug delivery as carriers for many types of cancer 12,13. Moreover, nanoparticles are used for drug delivery system which were extended for directing nanoparticles by using an external magnetic field in particular places due to accurate treatment 14. Therefore, it is assumed that Reactive Oxygen Species (ROS) produced by nanoparticles have a bacteriostatic potency without damaging eukaryotic cells 15,16.

The aim of this study was to find a way to prevent drug resistance by using lower doses of antibiotics to treat the bacterial infections.

## Materials and Methods

The reference strain, *E. faecalis* (ATCC 11700), was used as the control strain in all steps for comparison purposes.

### Preparation of culture medium

In total, 20 culture-positive specimens of *E. faecalis* were collected from patients at Tehran University of Medical Sciences and cultured on Bile Esculin Agar (Merck, Germany) 17,18.

### Kirby-Bauer Susceptibility Test

In order to test the resistance of *E. faecalis*, clinical-positive specimens of these bacteria were cultured on Mueller Hinton agar medium (Merck, Germany). Disk diffusion was performed using Kirby-Bauer method (Clindamycin 2 *μg*, Oxacillin 1 *μg*, Erythromycin 15 *μg*, Cefotaxime 30 *μg*, Ceftazidime 30 *μg*, Tetracycline 30 *μg*, Chloramphenicol 30 *μg*, Vancomycin 30 *μg* disks, Mast Group Ltd Company, UK). Incubation was performed for 24 *hr* at 37*°C* with 5% CO_2_ (Semi-aerobic conditions). The isolate with higher resistance was selected for further study.

### Preparation of nanoparticle (NP) suspension

Fe_2_O_3_ nanoparticles (Purity over 99.7%) with 10–50 *nm* range size were purchased from US NANO. Nanoparticles stock solution was prepared by suspending one gram of nanoparticles into 100 *ml* sterile medium and dispersion was done by Electro sonic system (Bandelier Sonorex RK 31H) for 35 *min*. The microbial tests and preparation of nanoparticle suspensions were performed simultaneously in order to reduce probable errors.

### Microbial suspension preparation

At first, bacterial cells were collected from BEA culture medium and were mixed in 10 *ml* Phosphate-Buffered Saline (PBS) in order to prepare samples with 0.5 McFarland Turbidity [1–1.5×10^8^ Colony-Forming Unit (CFU)] and the accuracy was measured by spectrophotometer (UNICO-2100; USA) at 620 *nm* wavelength range and absorbance was set at the range from 0.08 to 0.1 *nm*.

### MIC test and bacteriostatic potency

The Clinical and Laboratory Standards Institute (CLSI) recommendations were used for Minimal Inhibitory Concentration (MIC) calculation of the sample in contact with the NP suspension. Gradient concentrations of Fe_2_O_3_NPs suspension, both NPs with bacteria, were prepared according to a conducted study by Khavarani *et al* 18,19.

### Nanoparticles impregnated discs preparation

Sterile blank discs (Crude) were placed in a plate, then nanoparticle suspension with desired concentration was poured into the plate, where the discs were not immersed, then incubated about 24 *hr* at room temperature until the suspension was completely absorbed by blank discs 20.

### DDST susceptibility test

The antibiotics to which the isolate showed resistance or semi-susceptibility were selected and assessed for DDST in Mueller Hinton agar medium with 20 *mm* center to be centered in the plate. Incubation was performed for 18 *hr* at 37*°C* and the inhibition zone from the edge of each disc was recorded 19.

## Results

### Kirby-Bauer Susceptibility Test

The isolated bacteria were resistant to Oxacillin and Ceftazidime, and semi-sensitive to Tetracycline and Chloramphenicol acid antibiotics (Table 1).

**Table 1.** Sensitivity of the *E. faecalis* to the applied antibiotics and in synergy with Fe _2_ O _3_ nanoparticles

	**ATCC11700**		**Clinical Sample**	

**Antibiotic**	***μg***	**Zone diameter (*mm*) Mast Disc**	**Zone diameter (*mm*) Mast Disc**	**Zone diameter (*mm*) DDST**
**Clindamycin**	2	10	0	12
**Oxacillin**	1	0	0	12
**Erythromycin**	15	25	23	NA
**Cefotaxime**	30	19	18	NA
**Ceftazidime**	30	17	0	13
**Tetracycline**	30	13	13	NA
**Chloramphenicol**	30	11	11	12
**Vancomycin**	30	25	20	NA

* (NA)-did not test.

### Preparation of the nanoparticle suspensions

**Figure 1.**
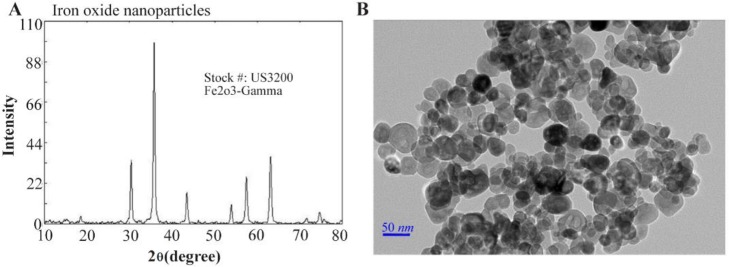
A) XRD of Fe_2_O_3_ nanoparticles, B) TEM of the Fe_2_O_3_ nanoparticles.

The X-ray powder diffraction (XRD) diagram of the commercial Fe_2_O_3_ powder in figure 1A demonstrates the proper purity due to lack of any impurity diffraction pattern. Figure 1B presents the corresponding morphology and particle size distributions of prepared Fe_2-_O_3_ powders by TEM microscopy.

### MIC test and bacteriostatic potency

**Figure 2.**
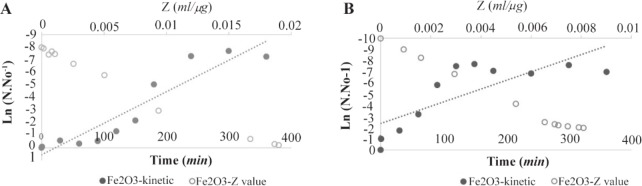
*E. faecalis* population coefficient sensitivity due to the time of investigation. A) ATCC11700 (MIC after 180 *min*)-B) Clinical sample (MIC after 120 *min*).

According to the results, the antimicrobial activity of Iron oxide nanoparticles suspensions against *E. faecalis*, the MIC for the isolated bacteria and ATCC-11700 strain against Iron oxide NPs were Z=78.125 *ml/μg*^−1^ and 39.0625 *ml/μg*^−1^, respectively. The sensitivity coefficient for *E. faecalis* against the nanoparticle suspension was calculated for each sampling period. The mean of bacterial coefficient sensitivity to the nanoparticles is shown in figure 2.

### DDST susceptibility test

The results in table 1 show higher antibacterial activity for Clindamycin, Oxacillin, and Ceftazidime due to visibility of the zone; however, it seems Chloramphenicol in synergy with the applied Fe_2_O_3_ did not increase the inhibitory zone significantly.

## Discussion

XRD and TEM images illustrate the appropriate crystal structure of synthesized Iron oxide NPs and have almost regular spherical shape in the size range of nano. Some bacteria have the potency in reducing metal oxide by mechanisms 15,16, therefore reducing the size of Iron NPs would not be a good idea for increasing toxicity of metal oxide 21; but nanoparticles with higher number of reactive groups on the surface like active sites for the formation of ROS which lead to oxidative stress could be good candidates 15,22.

Many studies reveal that in aquatic system, the antimicrobial activity of metal oxide compounds was mainly caused by soluble ions, and has effect on reduced cellular function due to aggregation of NPs in aqueous medium 23. But at efficient concentration, NPs at dilution condition can be more toxic for cells than the metal ionic form which is described as a nanotrojan horse type of mechanism 22.

According to figure 2, NPs inhibitory properties against *E. faecalis* were enhanced by increasing nanoparticles concentration. But higher concentration of nanoparticles leads to slight growth; so, Iron oxide nanoparticles had no bactericidal effect on tested strains.

Previous studies represent that, if the environmental pH is lower than the pH of NPs, the surface of NPs could be positively charged and vice-versa 24. Theoretically, the pH values of Fe_2_O_3_ NPs have been calculated to be 5–7. Due to lack of significant difference between both pH values (pH of microorganism is about 2–4), this opinion could not be justifiable.

The results express that Chloramphenicol in synergy with NPs could not increase inhibitory effect significantly (Table 1). According to the given explanations, surface charge of bacteria is reduced due to repulsion 25. Therefore, greater concentration of NPs due to this electrostatic repulsion force cannot be a suitable option to overcome this inhibitory force 24.

## Conclusion

Based on our findings, it can be inferred that Iron oxide nanoparticles suspension in different concentrations has growth inhibitory effects against bacteria that cause nosocomial infections, but at higher concentrations than the MIC, presumably it can make adaption to Fe^2+^ ions which could be used as a source of energy in metabolic pathway.

The use of bacteriostatic potency of these NPs against bacteria suspensions with certain concentration in combination with antibiotics can be a good option for inhibition of the bacterial infections in medical domains.
